# Feasibility of Acquiring Neuroimaging Data from Adults with Acquired Brain Injuries before and after a Yoga Intervention

**DOI:** 10.3390/brainsci13101413

**Published:** 2023-10-05

**Authors:** Jaclyn A. Stephens, Denny Press, Jennifer Atkins, John R. Duffy, Michael L. Thomas, Jennifer A. Weaver, Arlene A. Schmid

**Affiliations:** 1 Department of Occupational Therapy, Colorado State University, Fort Collins, CO 80524, USA; jen.weaver@colostate.edu (J.A.W.); arlene.schmid@colostate.edu (A.A.S.); 2 Molecular, Cellular and Integrative Neuroscience Program, Colorado State University, Fort Collins, CO 80521, USAmichael.l.thomas@colostate.edu (M.L.T.); 3 Adaptive Yoga Specialist, LLC, Fort Collins, CO 80528, USA; jennifer@adaptiveyogaspecialist.com; 4 Psychology Department, Colorado State University, Fort Collins, CO 80523, USA; john.duffy@colostate.edu

**Keywords:** Hatha yoga, acquired brain injury, resting-state functional magnetic resonance imaging, functional near-infrared spectroscopy, feasibility study

## Abstract

Background: To date, no one has prospectively evaluated yoga intervention-induced changes in brain structure or function in adults with acquired brain injuries (ABI). Thus, this study was conducted to test the feasibility of acquiring neuroimaging data from adults with ABI before and after a yoga intervention. Methods: This was a single-arm intervention feasibility study that included 12 adults with chronic (i.e., greater than 6 months post-injury) ABI and self-reported limitations in balance. Neuroimaging data were acquired before and after yoga. The yoga intervention was completed once per week for eight weeks. Feasibility objectives and benchmarks were established a priori. Results: Most feasibility objectives and benchmarks were achieved. The goal of recruiting 12 participants was successfully achieved, and 75% of participants were retained throughout the study (goal of 80%). All imaging feasibility benchmarks were met; rs-fMRI and fNIRS data were acquired safely, data were of acceptable quality, and data pre-processing procedures were successful. Additionally, improvements were detected in balance after yoga, as group-level balance was significantly better post-yoga compared to pre-yoga, *p* = 0.043. Conclusions: These findings indicate it is feasible to acquire neuroimaging data from adults with ABI before and after a yoga intervention. Thus, future prospective studies are warranted.

## 1. Introduction

Each year, approximately 2.9 million Americans sustain traumatic brain injuries (TBI) that result in emergency department visits, hospitalizations, and death [1]. TBIs occur when an external mechanical force affects the head, neck, or body and causes rapid acceleration and deceleration of the brain within the skull, resulting in damage to brain tissue. ABI is a broader term that includes TBI and other forms of brain tissue damage, such as damage induced by lack of oxygen (anoxia), reduced blood flow (cerebral vascular accident or stroke), brain tumors, or other non-mechanical methods of injury (e.g., poisoning). Although there are many treatment strategies in the early weeks and months after ABI, millions of individuals live with residual disability [2,3,4]. This residual, chronic disability often includes significant physical impairments and ongoing disruptions in brain function [5]. One particularly impactful physical impairment is poor balance, which is associated with increased fall risk, limited community integration, and reduced quality of life [6,7,8].

Fortunately, community-based and holistic interventions, such as hatha yoga, may be effective in addressing balance in adults with ABI, as previous work has shown its effectiveness in aging [9] and other neurological populations [10]. Hatha yoga incorporates movements that require balance, can be adapted for individual needs, and does not require physician or insurance authorization. Currently, a few quantitative studies investigating yoga for individuals with brain injury have found evidence of improved balance after yoga [11], and other studies (including qualitative studies) have found yoga-induced improvements in outcomes such as quality of life [12], community integration [13], and overall physical functioning [14] in adults with brain injury. However, the neural underpinnings, or changes in brain structure or function, of functional improvements in specific capacities, such as balance, after yoga are largely unknown. To date, no one has investigated if yoga-induced improvement in balance or other functional capacities is supported by changes in brain structures or function in adults with ABI.

Importantly, there is compelling evidence that yoga can improve brain function—specifically functional connectivity of neural networks as measured with functional magnetic resonance imaging (fMRI)—in healthy adults (see review: [15]). There is also evidence that yoga breathing can positively influence cortical oxygen metabolism in healthy adults, as measured by functional near-infrared spectroscopy (fNIRS; [16]). Thus, it is possible that similar improvements could be detected in adults with ABI, but to the best of our knowledge, this has not been tested.

Because ABI elicits damage in brain regions and neural networks and is associated with disability and impairment [5,17,18], it can be more difficult to study adults with ABI than healthy individuals. Individuals with ABI may have more difficulty participating in longitudinal intervention studies due to transportation issues, medical conditions (e.g., epilepsy) that interfere with research appointments, or difficulty engaging in intervention activities that do not accommodate their abilities. Therefore, feasibility studies should be conducted prior to large-scale intervention studies. Of course, multiple research teams have successfully conducted intervention studies with individuals with TBI or ABI (e.g., [19,20], but potential barriers to participant recruitment and retention must be evaluated prior to conducting large-scale work when new protocols are used.

The feasibility of neuroimaging methods with individuals with ABI also requires additional consideration. Maintaining participant safety is a principal objective for all studies, and individuals with ABI may have additional safety needs. For example, individuals with ABI often have limited mobility and use mobility devices (e.g., canes or walkers) for ambulation [21]. Research studies conducted outside of medical institutes may not have MRI-compatible devices to support ambulation to and from the scanner, which poses a significant safety or fall risk. Additionally, individuals with ABI are more likely to have contraindications for scanning (e.g., metal implants), so careful screening procedures that consider participants’ cognitive abilities when answering screening questions are essential. Acquiring high-quality MRI data can also be challenging. Participants with ABI may be more likely to move during data acquisition or have changes in cortical blood flow from their injury that confounds the evaluation of the blood-oxygen-level-dependent (BOLD) signal [22], which is a proxy measure for neural activity. These can negatively influence individual data quality and/or reduce the ability to generate group-level data. Again, there are known solutions for these limitations, yet researchers must first confirm that they can acquire quality MRI data with planned procedures for future studies.

Newer neuroimaging techniques, such as fNIRS, allow for the evaluation of brain activity with fewer restrictions. However, there are few established methods for fNIRS data acquisition and analysis—particularly for portable fNIRS devices [23]—which threaten the usefulness of studies conducted with both healthy individuals and patient populations. Moreover, there are additional safety and data quality components that must be considered when conducting research with individuals with ABI. Finally, adaptations to the administration of performance-based measures, such as balance assessments, to permit simultaneous fNIRS acquisition may influence performance data. Thus, solutions that preserve both behavioral and neural data need to be developed to optimize study procedures.

In summary, there is some evidence that yoga can improve functional capacities, such as balance, in adults with ABI [11], but prospective yoga-induced changes in brain function have not been evaluated. Thus, this study was completed to assess the feasibility of acquiring neuroimaging data from adults with ABI before and after a hatha yoga intervention.

## 2. Materials and Methods

Study Design and Location: A single-arm longitudinal intervention feasibility study was completed, and the study protocol is available on clinicaltrials.gov, protocol # NCT05895084. Neuroimaging data were acquired within university laboratories by trained researchers at assessment visits that occurred 1–2 weeks before and after the hatha yoga intervention. The hatha yoga intervention was completed once per week for eight weeks in a classroom on a college campus. Colorado State University’s institutional review board approved all study procedures (Protocol #1799), and all participants provided informed written consent. Seven primary feasibility objectives and benchmarks were identified a priori; see Table 1 in Results.

Participants: Prospective participants were recruited via email list serves, newsletters, flyers, and word-of-mouth. To ensure safe delivery of the hatha yoga intervention, the targeted sample size was 12 participants. Phone screenings were completed to determine study eligibility. Participants were included in the study if they had a TBI or ABI that occurred ≥ 6 months prior and had self-reported balance limitations. Participants were also screened for significant developmental or neurological conditions that were diagnosed before their ABI, and no participants had other significant conditions. Individuals were excluded if engaged in non-adapted yoga. Of the 12 participants included in the yoga intervention, we were able to include 6 for MRI methods (detailed below), given the availability of pilot study funds. Participants were screened multiple times for MRI safety to detect contraindications (e.g., metal plates in the head, claustrophobia, etc.) and were included in the hatha yoga intervention even if they could not complete MRI scans because other study data (e.g., fNIRS data) could be acquired. Participants provided written informed consent and were reimbursed at a rate of $20 for each assessment visit.

Yoga Intervention: Hatha yoga was delivered by an adaptive yoga specialist and trained aides once/week for eight consecutive weeks (eight total sessions). Yoga was delivered in a standardized progression and included mindful breathing with functional movements, breathwork, mantras, progressively challenging yoga postures (sitting in chairs, standing, and floor), and guided relaxation/meditation (see Supplement Figure S1). Each yoga session lasted one hour, was in person, and was delivered in a group format. Adaptations were incorporated to support successful engagement in each class and included modifications to poses, hands-on assistance to support participants in moving and holding poses, and the use of chairs/props to allow participants to safely and successfully complete postures.

### Measures

*Resting State Functional Magnetic Resonance Imaging (rs-fMRI):* Functional scans sensitive to the T2-weighted BOLD signal were collected using a gradient echo pulse sequence with multiband and echo-planar imaging options (Repetition Time (TR) = 800 ms; Time to Echo (TE) = 38 ms; flip angle = 52°; Field of View (FOV) = 210 mm; matrix size = 84 × 84; in-plane resolution = 2.5 mm; slice thickness = 2.5 mm; slices = 54; slice spacing=0; multiband factor = 6) using a 3 Tesla Siemens Skyra Magnetom and a 32-channel head coil.

Software from Analysis of Functional NeuroImages (AFNI) was used to pre-process the structural and functional images. Images were processed using a pipeline that includes segmentation, distortion correction, de-spiking, alignment and co-registration of the functional images to the structural images, detection of outliers, and blurring. A concern related to all rs-fMRI datasets is excessive movement, which can produce poor connectivity maps. That is, if a participant is moving too much during scanning, this can produce invalid correlations between the seed and other voxels in the brain images. As noted above, a feasibility objective for rs-fMRI data was to determine whether pre-processing algorithms that align the rs-fMRI volumes in the time series can produce acceptable quality indices for 80% of the data points. Additionally, we compared the data from this study with 51 healthy subjects from a separate study completed using the same MRI scanner to compare quality indices. The quality index is operationalized as one minus the Spearman correlation coefficient of each image in the time series with the median value (AFNI 3dTqual). Poor quality is defined as quality index values that are 3.5 times the median absolute deviation (3.5 * MAD). As an additional quality metric, six motion parameters—three translations and three rotations—were extracted for each participant and each volume using 3dvolreg in Analysis of Functional NeuroImages (AFNI) [24]. The rotations were converted to distance. Framewise displacement (FD) was then calculated for each volume by taking the Euclidean distance of the translations and rotations [25]. The following formula was used:

FD = |dx| + |dy| + |dz| + |r_x| + |r_y|+ |r_z|

where dx, dy, and dz are the absolute displacement values for translations in the x, y, and z directions, and r_x, r_y, and r_z are the absolute displacement values for rotation around the x, y, and z axes, respectively.

Another feasibility objective was to use individual rs-fMRI scans to produce a valid group-based motor connectivity map from the precentral gyrus (i.e., primary motor cortex) seed. In particular, the connectivity map should show graded patterns of activity rather than random pixelation. To produce this map, AFNI’s 3dDeconvolve tool was used. A general linear model (GLM) was applied to each participant’s co-registered functional images (ignoring censored values). The GLM analysis incorporated covariates accounting for linear, quadratic, cubic, and quartic drift and six motion parameters, as well as the seed (precentral gyrus) time series. AFNI’s 3dttest++ function was then used to produce seed-based connectivity maps. The validity of this map was judged qualitatively.

**Figure 1:**
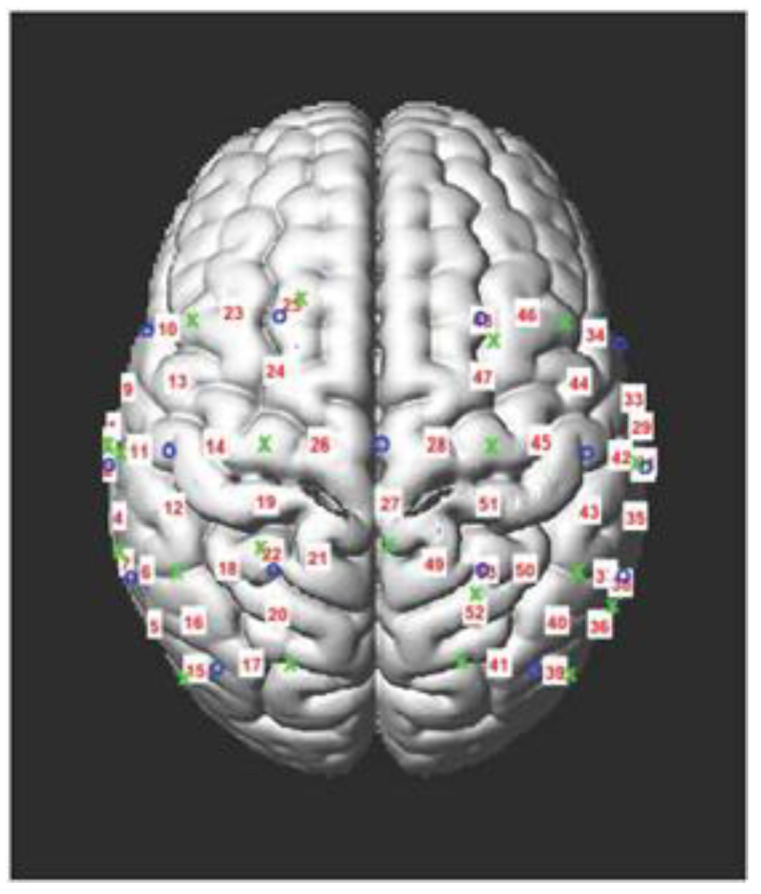
fNIRS Data Acquisition. fNIRS optodes—light sources and light detectors—were placed over bilateral motor cortices and inferior parietal sulci to form 53 total channels (i.e., the intersection of a light source and detector), as depicted in this figure.

*Functional Near-Infrared Spectroscopy (fNIRS) Acquisition with Concurrent Balance Testing:* Balance performance and neural activity were evaluated simultaneously using a portable fNIRS device, the NIRSport2 (nirx.net), which can accommodate gross motor movements in more naturalistic environments. Participants were fitted with an appropriately sized cap that positioned optodes (i.e., near-infrared light sources and detectors) over bilateral motor cortices and inferior parietal sulci, as these regions are associated with balance [26]; see Figure 1 for optode placement.

**Figure 2:**
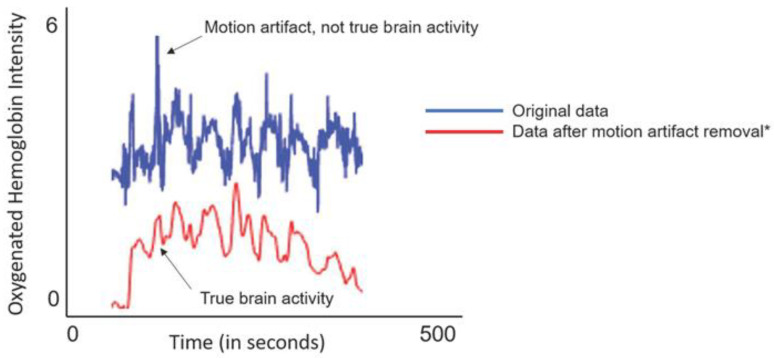
Detecting and Removing Motion Artifacts from fNIRS Data. This figure illustrates how the motion artifact regression algorithm (MARA^15^) is applied to raw fNIRS data. Visual inspection of raw fNIRS data (blue line) shows clear motion spikes or artifacts. During the pre-processing step of temporal processing, motion artifacts are detected and regressed from the data, resulting in data that depict true brain activity (red line). * These data have been generated for illustration purposes; they do not represent real participant data.

Once the cap was placed, a signal optimization step was completed to examine all 53 channels, the intersection of light sources and detectors, to assess if high-quality data could be acquired. This required that each light source was emitting an adequate amount of near-infrared light for absorption in participants’ superficial cortical structures and that the refracted light could be measured, in optical density, by the detectors. If any channel did not reach an acceptable or excellent level, high-quality fNIRS data could not be acquired. Acceptable and excellent levels reflect how well light passes through tissues and is measured in millivolts (mV); excellent values are those greater than 3 mV, acceptable values are between 0.5 mV and 3 mV, and critical values (when data should not be acquired) are below 0.5 mV. Additionally, signal quality was assessed, and high-quality data could be acquired when signal quality was at acceptable or excellent levels. Signal quality was evaluated using a coefficient of variance—the ratio between the standard deviation of the raw signal—as calculated over 1.5 s of data. Excellent coefficient of variance values is less than 2.5%; acceptable values are between 2.5% and 7.4%, and critical values (when data should not be acquired) are at or above 7.5%. All fNIRS data were acquired using Aurora software (nirx.net) and pre-processed using Satori software (nirx.net). Pre-processing included the conversion of optical density data to hemoglobin metrics of oxygenated hemoglobin (HbO), deoxygenated hemoglobin (HbR) and total hemoglobin (HbT), spatial registration to the head probe, and temporal processing. During temporal processing, a motion artifact detection and regression process was used to detect and remove motion artifacts; see Figure 2.

**Figure 3:**
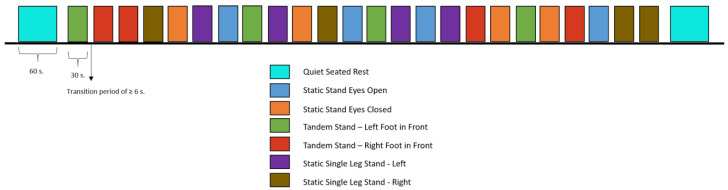
**Block Design of Balance Items**. Six items were used from the Berg Balance Scale (BBS) [27,28] to permit simultaneous fNIRS acquisition. The duration of each item was 30 s, and items were presented four times each in a randomized block design for 24 trials. Prior to the start of balance items and at the end of all balance items, participants completed a 60 s quiet seated rest period (turquoise rectangles).

Baseline fNIRS data were obtained during 60 s periods of quiet, seated rest at the beginning and end of balance tasks. Task-dependent neural data were acquired during each balance task. Six balance tasks from the Berg Balance Scale (BBS; [27])—a measure that has been validated for ABI populations [28]—were adapted and repeated for four trials at a duration of 30 s per trial in a randomized block design, for a total of 24 trials. Task order was randomly generated for each participant to prevent neural habituation using stimulus presentation software, PsychoPy [29], which interfaced with Aurora via a lab-streaming layer to segment the fNIRS data acquisition file with trial markers. BBS tasks included: static stand with eyes open, static stand with eyes closed, tandem stand with the left foot forward, tandem stand with the right foot forward, static single leg stand on the left leg, and static single leg stand on the right leg; see Figure 3 for an example block design. During balance tasks, participants wore a gait belt and were supported, as needed, by the study PI.

Balance Performance Scoring and Statistical Analysis: Due to adaptations that permitted fNIRS data acquisition, standardized BBS scoring criteria could not be used. Thus, balance was videotaped and evaluated using Functional Independence Measure (FIM) scoring criteria, where scores range from Dependent (1) to independent (7) to indicate the amount of assistance participants needed during each balance task [30]. Performance for each trial of the six tasks was documented and assigned a FIM score. Next, an average score was calculated for each balance task (e.g., static stand with eyes open). A total score was calculated by averaging the average scores from each of the six balance tasks. Finally, a Wilcoxon signed-rank test was used to compare group pre-yoga and post-yoga total scores with SPSS version 26 software.

## 3. Results

Overall, the study procedures were feasible; however, not all benchmarks were fully met; see Table 1.

*Recruitment and Retainment of Participants:* Over six weeks, 12 adults (5 males, mean age = 47.30, SD = 15.78) were recruited for pre- and post-yoga assessments and 8 weeks of in-person yoga classes; see Table 2. All 12 participants completed pre-yoga assessments. Six of the twelve participants were both eligible and agreeable to MRI scanning; four were not assessed for eligibility due to limited funding, and two were eligible for MRI but declined due to claustrophobia and/or dislike of MRIs. Nine of the twelve (75%) participants completed all or nearly all yoga sessions (average number of sessions attended 6.60, SD = 1.90). Two participants had scheduling and transportation issues that prevented regular yoga attendance, and one participant withdrew from the study due to an unrelated illness. Nine participants (83%) completed post-yoga assessments, but only seven completed all post-yoga measures. Two participants only completed post-yoga self-report measures due to pre-yoga fNIRS acquisition issues (*N* = 1) and a scheduling conflict that prevented post-yoga fNIRS acquisition (*N* = 1). Finally, 5 of the 6 participants who completed pre-yoga MRI scans also completed post-yoga MRI scans. No adverse events were reported during yoga.

*Safety of rs-fMRI Data Acquisition:* MRI safety screening detected all contraindications for MRI scans, and no adverse events occurred during pre- or post-yoga scans. Three of the six participants had mobility impairments, but MRI-compatible devices were not available during scanning. As such, physical assistance was provided by the study PI to help participants ambulate to and from the scanner and to transfer from seated to supine and supine to seated for scans. No participants sustained falls or near falls during scanning procedures. One participant experienced mild shortness of breath due to lying flat during the pre-yoga MRI assessment, but the study team monitored closely and paused the scan. This participant returned for the post-yoga MRI assessment and did not experience shortness of breath during that scan.

*Data Quality Indices of Pre-Processed rs-fMRI Images:* All subjects retained over 80% of time points. The highest retained 100%, and the lowest was 86.4%, all well within the acceptable range. We also compared the 3dTQual statistics for the 6 MRI subjects to the 3dTQual statistics for 51 healthy controls from the other study (used as a normative sample). We z-scored the 6 subjects’ values in comparison to 51 subjects and then calculated their percentile. The percentiles were 90th, 18th, 21st, 17th, 15th, and 28th. Since no value exceeds 95%, the data supports the adequacy of alignment in this sample. Additionally, the FD analysis produced FD figures with a range of 0.13 to 0.74 mm with a mean FD of 0.299 (just below a standard cutoff [25]). Two subjects had FD above the cutoff (0.74 and 0.31), while the other four subjects were well below.

**Figure 4:**
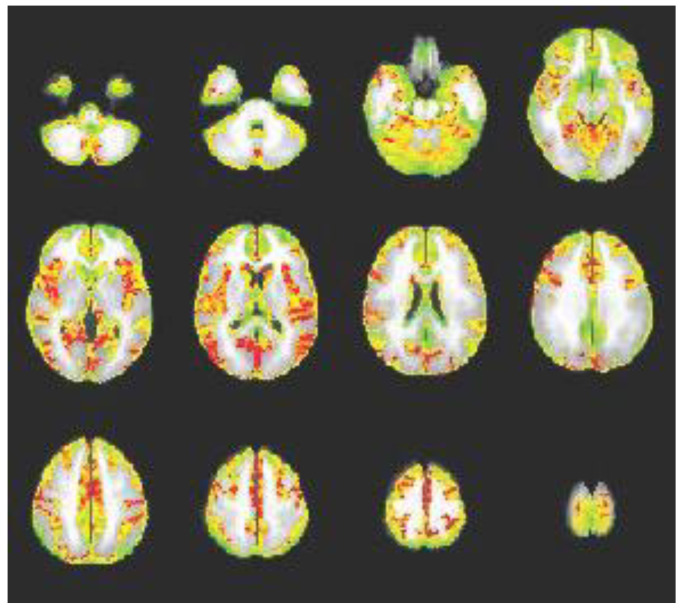
**Group Seed-Connectivity Map for Precentral Gyrus**. This figure depicts group-level connectivity between the precentral gyrus and multiple brain regions (in red). Importantly, the pattern is graded, showing transitional areas of connection rather than random pixelation.

*Group-Based Connectivity Map:* The group connectivity map was deemed a valid connectivity map, as connectivity (red areas) can be observed between the precentral gyrus and multiple other brain areas that are associated with motor function: bilateral insula, superior parietal lobule, paracentral lobule, posterior cingulate, superior and middle prefrontal areas, occipital areas, and cerebellar areas; see Figure 4. Importantly, the pattern is graded, showing transitional areas of connection rather than random pixelation; transitional areas of connection are indicative of strong functional connections.

*Safety and Quality of Functional Near-Infrared Spectroscopy (fNIRS) Data:* Eleven of the twelve participants attended pre-yoga visits for fNIRS data acquisition; one person did not attend due to illness and an inability to reschedule prior to start of yoga. Of these 11 individuals, fNIRS data were acquired from 10 participants (90.90%). Adequate signal quality levels could not be obtained from one participant who had thick, coarse hair that prevented optodes from reaching her scalp. Of the 10 participants with pre-yoga fNIRS data, post-yoga fNIRS data were acquired from 7 (see above for dropout details). Acceptable signal optimization levels were reached, and all motion artifacts were successfully detected and removed in 10 (90.9%) participants during the pre-yoga assessment and in 7 (100%) participants during the post-yoga assessment.

**Figure 5:**
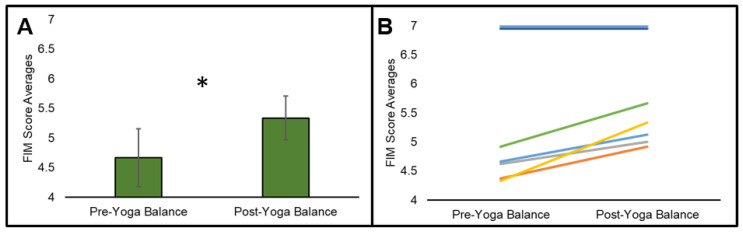
Yoga-Induced Balance Improvements. At a group level (**A**), post-yoga balance was significantly better than pre-yoga balance, even with modification to the balance measure and scoring. However, at an individual level (each participant is represented by a different colored line) (**B**), changes in balance could not be detected in 2/7 participants (both with TBI) due to FIM ceiling effects, although the majority had measurable improvements. Error bars represent standard error, * indicates *p* < 0.05.

*Balance Performance Results:* There were significant yoga-induced balance improvements, as post-yoga total scores (Median = 5.33, SE = 0.37) were significantly higher than pre-yoga total scores (Median = 4.67, SE = 0.49), *p* = 0.043; see Figure 5.

## 4. Discussion

Yoga may improve functional capacities, such as balance and brain function, in adults with ABI. In order to test this hypothesis, feasibility objectives were developed and assessed. All feasibility objectives, but the first, were fully met, and these findings provide important insights. The partial achievement of the recruitment and retainment objective highlights the limited transportation options available in the small city where this study was conducted. Participants who could drive independently or be driven by care partners had more regular attendance than participants who attempted to use public transportation. Additionally, positive characteristics of the intervention were illuminated. Specifically, no participant reported missing yoga because of an inability to engage in yoga class activities. This indicates that the yoga intervention, which included varied adaptations and support, provided a ‘just right challenge’ for these participants.

Another insight was that some aspects of acquiring MRI data were challenging. The university’s MRI scanner is located in a non-medical research facility, so MRI-compatible walkers and canes were not available. For future work, we have acquired MRI-compatible mobility devices to support the broader inclusion of participants. Still, study outcomes indicated that MRI study procedures were safe and could generate group-level data. Importantly, group-level data can show functional improvements in brain structure and function from other interventions for adults with ABI (e.g., [31]. Thus, the methods tested in this feasibility study have the potential to demonstrate how a hatha yoga intervention elicits neural improvements in adults with ABI.

A final insight is that both high-quality fNIRS data could be acquired and pre-processed with the included methods, which were novel and not previously tested. Further, as in previous work [11], post-yoga balance improvements were observed. This likely indicates that yoga-induced balance improvements are robust and can be detected even when balance testing and scoring are modified to permit simultaneous neuroimaging. Arguably, the success in preserving both balance and fNIRS data is the most impactful outcome of this feasibility study.

*Study Limitations:* Despite achieving nearly all of the feasibility benchmarks, this study had some notable limitations. First, all participants were recruited from a single small city, which limits generalizability and had heterogeneous forms of ABI. Additionally, some participants experienced scheduling and transportation issues, which limited participation. In future studies, we should consider requesting information from participants (e.g., via qualitative interviews) about their scheduling and transportation needs. This will allow us to adjust class times to meet their needs and/or offer virtual attendance options (provided that this does not pose a risk to safety). Additionally, in future studies to improve access to yoga classes, we should seek additional funds to pay for transportation for participants who cannot drive or do not have caregivers available to drive them to classes. As another limitation, pre-study activity levels were not formally assessed, nor did the research team consistently monitor activity levels during the study. This could have influenced findings and will need to be explicitly measured in future work. Additionally, we only had funding to complete MRI methods with 6 of the 12 participants and lost 1 participant to follow-up, so our MRI feasibility data may not be fully representative of our sample or the ABI population. We also had some issues with MRI data quality due to participant movement inside the scanners. In future work, this could be remedied by using a mock scanner and providing additional training to participants to help them understand the importance of eliminating or reducing head movement during scans. One final limitation was the use of FIM scoring to evaluate changes in balance performance. Ceiling effects were observed, which is a well-documented limitation of the FIM [32] despite visible but unquantifiable improvements in stability and balance post-yoga. In future studies, inertial sensors or force plates may be beneficial for quantifying improvements in balance quality [33].

## 5. Conclusions

Hatha yoga has the potential to improve functional capacities, such as balance and brain function, in adults with ABI. Importantly, we observed improved balance after yoga despite having a small sample size and modifying our balance assessment procedures to permit simultaneous neuroimaging. Still, more research is needed to test for prospective yoga-induced changes in brain function, and examination of brain function may require more homogeneity in participant ABI injury type. Nevertheless, this study was completed to assess the feasibility of neuroimaging before and after a yoga intervention and demonstrated successful achievement in acquiring safe and high-quality neuroimaging data. These results support the use of neuroimaging before and after yoga in future pilot study designs with adults with ABI.

## Figures and Tables

**Table 1:** Feasibility Objectives, Benchmarks, and Outcomes.

Feasibility Objective	Feasibility Benchmark	Study Outcome
Recruit and retain adults with ABI for a longitudinal hatha yoga intervention with neuroimaging.	Recruit 12 adults with ABI Retain ≥ 80% (~10 of 12) over the course of the study.	√ Recruited 12 adults X Retained 75%
Safely acquire rs-fMRI data in participants with mobility limitations.	Detect 100% MRI contraindications during screening. Sustain zero instances of adverse events (e.g., pain or significant anxiety) or falls during data acquisition.	√ Detected all contraindications √ Zero adverse events or falls
Pre-process rs-fMRI and demonstrate acceptable rs-fMRI data quality indices.	Achieve time series quality indices < 3.5 * MAD in ≥ 80% of data time points. Observe similar quality indices as achieved in a normative sample. Achieve acceptable FD in the majority (4/6) participants.	√ Quality indices were observed in 86.4–100% of data time points. When compared to a normative sample, quality indices z-scores did not exceed 95%. Acceptable FD was reached in 4/6 participants.
Produce a valid group-based connectivity map from the precentral gyrus seed.	Complete visual inspection of the group seed-based connectivity map for the precentral gyrus and confirm strong connections between the premotor areas and other brain regions associated with motor function.	√The connectivity map is valid as visual inspection shows graded, transitional areas of connection rather than random pixelation.
Safely acquire fNIRS data during concurrent balance testing in participants with mobility limitations.	Have zero instances of adverse events (e.g., pain or anxiety) or falls during data acquisition.	√ Zero adverse events or falls
Acquire high-quality fNIRS data and successfully pre-process those data	Reach acceptable signal optimization and quality levels in ≥ 80% of participants assessed. Successfully detect and remove motion artifacts from data in ≥ 80% of participants assessed.	√ Acceptable signal and quality in data and all motion artifacts detected and removed in 90.9% of participants pre-yoga and 100% post-yoga
Detect yoga-induced improvements in balance with a modified balance measure when balance is assessed with simultaneous fNIRS.	Show a statistically significant improvement in post-yoga balance compared to pre-yoga balance performance.	√ Significant improvement in balance was detected while using a modified balance, as group-level improvements were observed post-yoga, *p* = 0.043.

Note: ABI = acquired brain injury, rs-fMRI = resting-state functional magnetic resonance imaging, fNIRS = functional near-infrared spectroscopy, MRI = magnetic resonance imaging, MAD = median absolute deviation, FD = framewise displacement.

**Table 2:** Participant Characteristics.

ID	Age at Study Onset	Sex	Education	Type of ABI	Time since ABI	# of Yoga Sessions
1	74	Female	Did Not Report	Stroke	13 years	8
2	55	Male	Doctorate	Severe TBI	2 years	7
3	66	Male	Some College	Stroke	2 years	8
4	29	Female	High School	TBI (severity not reported)	4.5 years	2
5	56	Male	Bachelor’s Degree	Anoxic Brain Injury	8 years	8
6	57	Female	Some College	Multiple Strokes	20+ years (exact # unknown)	7
7	29	Female	Some College	TBI (severity not reported)	9 years	Withdrew from study
8	34	Female	Some Graduate	TBI (severity not reported)	3.5 years	6
9	37	Male	Some College	Multiple Concussions/mTBIs	1.5 years since the most recent	8
10	30	Male	Some Graduate	Multiple Concussions/mTBIs	13 years since the most recent	7
11	33	Female	Bachelor’s Degree	Hydrocephalus	6 years	Withdrew from study
12	40	Female	Master’s Degree	Multiple Concussions/mTBIs	1 year since the most recent	5

Note: ABI = acquired brain injury, TBI = traumatic brain injury, mTBI = mild traumatic brain injury.

## Data Availability

Data are available upon request; please contact the corresponding author, Dr. Jaclyn Stephens.

## Supplementary Materials

The following supporting information can be downloaded at: https://www.mdpi.com/article/10.3390/brainsci13101413/s1, Figure S1: Example of Single leg balance, tree pose/vrksasana with modification to support performance; Table S1: Weekly yoga activities with reason and benefit.

## Clinical Trial Registration

This study is available on clinicaltrials.gov, NCT05895084.
