# Supplementary material for: Feasibility of Acquiring Neuroimaging Data from Adults with Acquired Brain Injuries before and after a Yoga Intervention
Source: Brain Sci. 2023 Oct 5;13(10):1413. doi: 10.3390/brainsci13101413 (PMC10605412; doi:10.3390/brainsci13101413)
Supplement: Supplementary file 1 [file brainsci-13-01413-s001.zip › brainsci-2602058-supplementary.pdf]

**Planned Yoga**	**Example**	**Reason/Benefit**
Week 1	All seated yoga with mantras (I am enough)	Yoga programming was developed to be progressively challenging, moving from seated, to standing, to getting to the floor. All movement was coordinated with breath. Mantras were used with each session
Weeks 2-8	Seated and standing yoga with mantras (I am enough)	
Weeks 5-8	Seated, standing, and floor yoga with mantras (I am enough)	
Breath work Weeks 1-8	Breath awareness during poses	Breath was coordinated with movement/poses, but specific pranayama were also used to encourage emotional regulation and relaxation
	Extended inhale/exhale during poses	
	Belly breathing with inhale and exhale in seated and corpse pose/savasana	
Seated poses Weeks 1-8	Face and neck movements with lateral and forward neck flexion	Relax face, stretch neck muscles
	Seated knees raised and bent, boat pose/paripurna navasana	Engage and strengthen core muscles
	Seated spinal flexion/extension, seated cat/cow/marjaryasana/bitilasana	Increase flexibility and strength of the neck, shoulders, and spine

Planned Yoga	Example	Reason/Benefit
Seated poses<br>Weeks 1-8	Seated crossing midline with upper body reaching	Coordination and communication between left and right hemispheres of the brain
	Seated leg lifts and forward fold/uttanasana	Calms mind, reduces stress, and blood pressure, compresses vagus nerve
	Seated wide open lunge, seated warrior II/virabhadrasana II	Strengthens and stretches leg and hip muscles
Standing poses<br>Weeks 2-8	Standing with feet hip distance apart, mountain pose/tadasana	Strengths spine and improves balance by addressing strength and stability of feet, ankles, thighs, and hips
	Lunge with hips squared forward, warrior I/ virabhadrasana I	Strengthens and stretches leg and hip muscles
	Chest and hip opener in a side lunge, warrior II/ virabhadrasana II	Strengthens and stretches leg and hip muscles, develops balance and stability, opens chest and lungs
	Forward bend from the hips, forward fold/ uttanasana	Stretnches the back of the body while at the same time calms mind, reduces stress, and blood pressure, compresses vagus nerve
	**Single leg balance, tree pose/vrksasana (see image below)**	Improves balance, strengthens core, increased awareness in body

Planned Yoga	Example	Reason/Benefit
Floor/mat poses and meditation Weeks 5-8	Mindfulness body scan	Enhances body awareness, decreases anxiety and reduces chronic pain
	Knees into chest, wind relieving/apanasana	Stretches and stabilizes pelvis and lower back
	happy baby/Ananda balasana	Stretches inner thighs, hips, groin, reduces lower back pain
	Seated cross legged, modified lotus pose/padmasana	Stretches hips, knees, and ankles and strengthens the upper back and spine, may increase circulation
	Supine relaxation, corpse pose/savasana	Calms the nervous system and the mind, reduces stress, allows time for the yoga practice to be integrated into the body

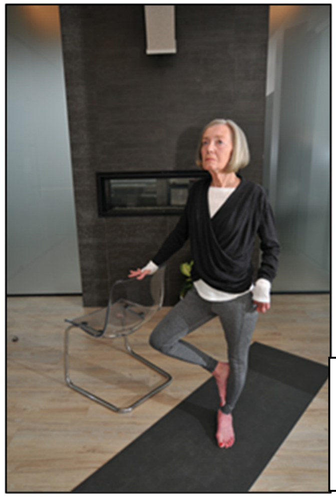

Image 1: Example of Single leg balance, tree pose/vrksasana with modification to support performance.
